# Gene profile analysis of osteoblast genes differentially regulated by histone deacetylase inhibitors

**DOI:** 10.1186/1471-2164-8-362

**Published:** 2007-10-09

**Authors:** Tania M Schroeder, Aswathy K Nair, Rodney Staggs, Anne-Francoise Lamblin, Jennifer J Westendorf

**Affiliations:** 1 Graduate Program in Biochemistry, Molecular Biology and Biophysics, University of Minnesota, 420 Delaware Street SW, Minneapolis, MN, USA; 2 The Cancer Center, and Department of Orthopaedic Surgery, University of Minnesota, MMC 806, 420 Delaware Street SW, Minneapolis, MN, USA; 3 Department of Orthopedic Surgery, Mayo Clinic, 200 First Street SW, Rochester, MN, USA

## Abstract

**Background:**

Osteoblast differentiation requires the coordinated stepwise expression of multiple genes. Histone deacetylase inhibitors (HDIs) accelerate the osteoblast differentiation process by blocking the activity of histone deacetylases (HDACs), which alter gene expression by modifying chromatin structure. We previously demonstrated that HDIs and HDAC3 shRNAs accelerate matrix mineralization and the expression of osteoblast maturation genes (e.g. alkaline phosphatase, osteocalcin). Identifying other genes that are differentially regulated by HDIs might identify new pathways that contribute to osteoblast differentiation.

**Results:**

To identify other osteoblast genes that are altered early by HDIs, we incubated MC3T3-E1 preosteoblasts with HDIs (trichostatin A, MS-275, or valproic acid) for 18 hours in osteogenic conditions. The promotion of osteoblast differentiation by HDIs in this experiment was confirmed by osteogenic assays. Gene expression profiles relative to vehicle-treated cells were assessed by microarray analysis with Affymetrix GeneChip 430 2.0 arrays. The regulation of several genes by HDIs in MC3T3-E1 cells and primary osteoblasts was verified by quantitative real-time PCR. Nine genes were differentially regulated by at least two-fold after exposure to each of the three HDIs and six were verified by PCR in osteoblasts. Four of the verified genes (solute carrier family 9 isoform 3 regulator 1 (Slc9a3r1), sorbitol dehydrogenase 1, a kinase anchor protein, and glutathione S-transferase alpha 4) were induced. Two genes (proteasome subunit, beta type 10 and adaptor-related protein complex AP-4 sigma 1) were suppressed. We also identified eight growth factors and growth factor receptor genes that are significantly altered by each of the HDIs, including Frizzled related proteins 1 and 4, which modulate the Wnt signaling pathway.

**Conclusion:**

This study identifies osteoblast genes that are regulated early by HDIs and indicates pathways that might promote osteoblast maturation following HDI exposure. One gene whose upregulation following HDI treatment is consistent with this notion is Slc9a3r1. Also known as NHERF1, Slc9a3r1 is required for optimal bone density. Similarly, the regulation of Wnt receptor genes indicates that this crucial pathway in osteoblast development is also affected by HDIs. These data support the hypothesis that HDIs regulate the expression of genes that promote osteoblast differentiation and maturation.

## Background

Histone deacetylases (HDACs) and histone acetyltransferases participate in chromatin remodeling and the regulation of gene expression. The opposing activities of these enzymes alter chromatin structure by either adding or removing acetyl groups from lysines in the amino-terminal tails of histones. The addition of acetyl groups to histones by acetyltransferases leads to the recruitment of co-activators and the relaxation of chromatin conformation that is necessary for transcriptional activation [1,2]. Conversely, removal of acetyl groups by HDACs results in a condensed chromatin structure that is restrictive to transcription. Mammalian HDACs are organized into four classes. Class I HDACs (1, 2, 3, and 8) display nuclear localization and ubiquitous tissue expression [3,4]. Class II HDACs (4, 5, 6, 7, 9, and 10) exhibit tissue specific patterns of expression, shuttle between the nucleus and cytoplasm, and are larger than class I HDACs [5]. Class III HDACs (Sirt1-7) require the coenzyme NAD^+ ^for enzymatic activity [6]. HDAC11 is the sole member of the new Class IV [4].

HDAC inhibitors (HDIs) broadly compromise the activities of class I, II and IV HDACs, albeit with varying efficiencies [7-9]. Natural and synthetic HDIs are divided into several structurally diverse classes including hydroxamic acids such as trichostatin A (TSA), short chain fatty acids such as valproic acid (VPA) and sodium butyrate (NaB), and benzamides such as MS-275 [10]. HDIs inhibit HDAC activity by blocking a channel that leads to the active site and a catalytic zinc ion [11]. In transformed cells, HDIs induce growth arrest, apoptosis, and/or differentiation via many mechanisms [7,10]. HDIs are currently in clinical trials as anticancer agents [10,12]; they are also established antiepileptic drugs [13] and potential treatments for inflammatory and cardiac diseases [14,15]. There are comparatively fewer data on the effects of HDIs on normal cells; however, the existing evidence suggests that normal cells are resistant to the anti-proliferation, pro-apoptosis and pro-differentiation effects of HDIs because their cell cycle checkpoints are intact [16,17].

We previously demonstrated that concentrations of TSA, MS-275 and VPA that were sufficient to induce histone H3 hyperacetylation in primary and MC3T3-E1 osteoblasts modestly increased cell proliferation and viability but had no effect on cell cycle progression [18]. More strikingly, HDIs accelerated the osteoblast maturation process by several days. Thus, short-term exposure to TSA accelerated the appearance of alkaline phosphatase activity and matrix mineralization as well as expression of type I collagen, osteopontin, bone sialoprotein, and osteocalcin genes in MC3T3-E1 cell cultures [18]. TSA, MS-275 and NaB also increased alkaline phosphatase activity in calvarial organ cultures [18]. Other studies showed that HDIs increase expression of genes associated with osteoblast maturation [19-22], enhance mineralization [21], block glucocorticoid-induced cell cycle arrest in osseous cells [23], and stimulate osteoblast differentiation of multipotent mesenchymal cells [24,25]. Suppression of HDAC1 or HDAC3 by RNA interference also accelerated osteoblast maturation [22,26]. These results suggest that the gene expression changes occur upon inhibition of HDACs and promote osteoblast terminal differentiation. In this study, we used an unbiased approach to identify osteoblast genes that are altered by HDIs within 18 hours to obtain a better understanding of the early pathways involved in accelerating the osteogenic phenotype.

## Results

### HDIs increase alkaline phosphatase activity during osteoblast differentiation

**Figure 1:**
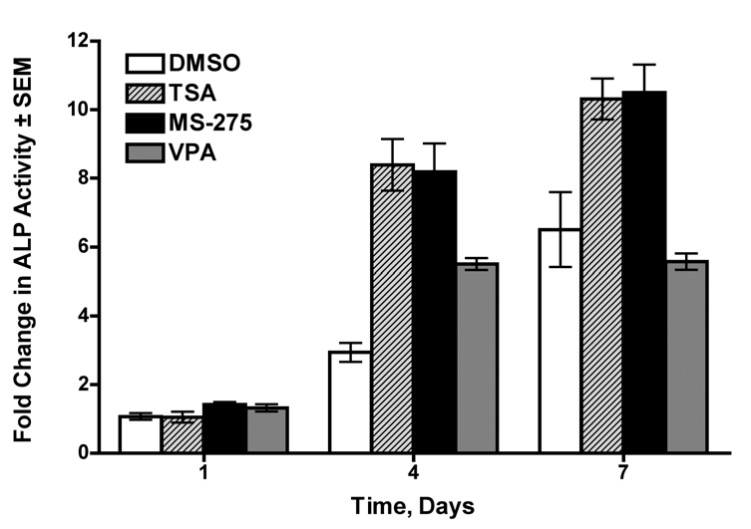
**HDI treatment accelerates the appearance of alkaline phosphatase activity in differentiating MC3T3 cells**. MC3T3 cells were cultured for the indicated times in osteogenic medium. The medium was changed three days with the HDIs or vehicle added only at day 0. Fold change in ALP activity is shown in relation to values obtained at the start of the culture (day 0). * denotes a statistically significant change of p < 0.01 by one-way ANOVA of the HDI-treated sample versus the DMSO-treated sample at that time point.

We previously demonstrated that HDIs accelerate and enhance alkaline phosphatase expression by MC3T3-E1 pre-osteoblasts after three days. By the second week, the HDI-exposed cultures expressed higher levels of genes associated with osteoblast differentiation (e.g. bone siaoloprotein, osteopontin and osteocalcin) than control cells [18]. The goal of this study was to identify genes that are affected early (within the first 18 hours) by several HDIs because they are more likely to be initiators or early regulators of the process. To demonstrate the differentiation potential of the HDI and vehicle-treated cells used in the microarray experiment, cells in parallel cultures were allowed to differentiate for up to seven days. During the differentiation process, lysates were taken at days 0, 1, 4, and 7 and alkaline phosphatase activity was measured. In the DMSO-treated cells, alkaline phosphatase activity increased steadily over time demonstrating that the cells were differentiating appropriately (Figure 1). Alkaline phosphatase activity also increased in the HDI-treated cells and was generally higher in HDI-exposed cells relative to vehicle-treated cells at days 4 and 7 (Figure 1). These results demonstrate that the MC3T3 cells used for microarray analysis differentiated appropriately and that the HDIs accelerated differentiation as expected from our previous studies [18].

### Gene expression profiles of HDI treated cells

To determine the molecular mechanisms whereby HDIs accelerate osteoblast maturation, we used microarray analysis to compare gene expression changes in MC3T3-E1 cells treated with HDIs or its vehicle, dimethylsulfoxide (DMSO). On the basis of our previous studies wherein we examined the expression changes in candidate genes after three days of HDI exposure [18], we hypothesized that HDI treatment at the beginning of differentiation would reprogram gene expression and accelerate the entire differentiation process. To identify the relatively early changes in gene expression that occur in response to HDIs in differentiating cells, we isolated RNA from MC3T3-E1 cells cultured in osteogenic medium and HDIs for only 18 hours. Corresponding probes were hybridized to Affymetrix GeneChip arrays and subjected to bioinformatics analyses.

At a false discovery rate (FDR) of 0.01 (99% confidence) relative to the vehicle (DMSO)-treated cells, 6117 genes were differentially expressed in the TSA-treated cells, 846 genes in the MS-275-treated cells, and 117 genes in the VPA-treated cells. The top 70 TSA-regulated genes, all altered by more than five-fold, are listed in Table 1. The genes altered more than two-fold by MS-275 and VPA are shown in Tables 2 and 3, respectively. The complete raw data sets have been deposited at the NCBI in the Gene Expression Omnibus data repository (GEO series record GSE9247) [27].

**Table 1:** Top 70 known genes altered by TSA in MC3T3-E1 cells

**Gene**	**Fold Change**	**Welch Test**
**Induced**		
Ubiquitin carboxy-terminal hydrolase L1	12.66	6.79E-06
Clusterin	12.58	1.80E-05
A kinase (PRKA) anchor protein (gravin) 12	9.431	1.06E-07
Abhydrolase domain containing 3	9.089	1.36E-05
Protease, serine, 35	9.058	3.48E-06
Glutathione S-transferase, alpha 4	8.408	3.35E-08
Amphiregulin	7.100	2.57E-06
Rab40b, member RAS oncogene family	6.904	1.16E-06
Thioredoxin interacting protein	6.865	1.61E-06
Apolipoprotein L, 2	6.604	0.001083
Laminin, alpha 1	5.616	1.70E-04
Solute carrier family 9, isoform 3 regulator 1	5.565	2.32E-05
Growth arrest and DNA-damage-inducible 45 alpha	5.422	3.67E-06
Insulin-like 6	5.418	6.71E-05
Thrombospondin 1	5.374	1.07E-04
Aldehyde dehydrogenase family 1, subfamily A7	5.359	4.73E-04
Erythrocyte protein band 4.1-like 5	5.266	6.39E-04
Hemoglobin alpha, adult chain 1	5.119	2.93E-06
Glutaminyl-peptide cyclotransferase (glutaminyl cyclase)	5.046	1.22E-05
		
**Suppressed**		
SMC4 structural maintenance of chromosomes 4-like 1	-5.192	5.96E-04
Kinesin family member 11	-5.266	1.07E-08
Lysyl oxidase	-5.284	1.66E-06
Antigen identified by monoclonal antibody Ki 67	-5.293	3.76E-04
Prostaglandin F receptor	-5.296	6.12E-04
Proteasome (prosome, macropain) subunit, beta type 10	-5.362	4.16E-04
Ubiquitin-conjugating enzyme E2C	-5.393	8.64E-06
Cyclin B1, related sequence 1	-5.408	1.21E-05
Cytoskeleton associated protein 2	-5.441	1.47E-07
Polo-like kinase 4	-5.456	1.03E-08
Chromodomain helicase DNA binding protein 3	-5.528	6.74E-04
Hyaluronan mediated motility receptor (RHAMM)	-5.547	5.41E-04
Kinesin family member 23	-5.605	2.30E-06
Epithelial membrane protein 3	-5.606	3.65E-05
Proteasome 26S subunit, ATPase 3, interacting protein	-5.623	5.01E-04
Cell division cycle 2 homolog A (S. pombe)	-5.693	1.43E-05
Formyltetrahydrofolate synthetase domain containing 1	-5.725	1.96E-04
Nucleolar and spindle associated protein 1	-5.780	1.81E-05
Centromere autoantigen A	-5.840	2.79E-04
Fibroblast growth factor 7	-5.856	6.42E-06
c-Fos induced growth factor	-5.931	1.99E-04
High mobility group box 2	-6.108	2.44E-08
Similar to G2/mitotic-specific cyclin B1	-6.166	1.07E-05
Cyclin A2	-6.311	3.35E-08
Cell division cycle associated 2	-6.395	3.47E-05
Calmodulin-like 4	-6.400	7.64E-04
Heat shock protein, alpha-crystallin-related, B6	-6.401	1.69E-04
Mannan-binding lectin serine protease 1	-6.413	3.19E-05
UDP glycosyltransferase 1 family, polypeptide A6	-6.498	8.67E-04
Calmodulin binding protein 1	-6.501	1.68E-08
Aurora kinase B	-6.531	1.74E-05
PDZ binding kinase	-6.548	3.16E-08
Minichromosome maintenance deficient 5	-6.613	6.08E-08
Interleukin 1 receptor-like 1	-6.641	6.40E-08
RAD51 associated protein 1	-6.877	9.26E-05
Centromere protein E	-6.911	3.17E-04
F-box only protein 5	-7.052	1.79E-07
Serine/threonine kinase 6	-7.090	1.10E-07
Stem cell growth factor	-7.199	6.95E-05
Proline 4-hydroxylase, alpha polypeptide III	-7.383	3.66E-07
Cell division cycle 20 homolog	-7.469	1.27E-04
Asporin	-7.618	6.15E-04
Glutathione peroxidase 7	-8.216	1.26E-07
Polo-like kinase 1	-8.301	9.95E-06
Sushi-repeat-containing protein	-8.701	5.38E-08
Ribonucleotide reductase M2	-9.796	2.00E-04
Chromosome condensation 1-like	-9.845	6.69E-04
Histone 1, H2ad	-10.21	4.61E-06
Procollagen, type III, alpha 1	-10.44	6.38E-04
Matrix metalloproteinase 13	-11.09	3.70E-05
Thymidine kinase 1	-16.96	2.88E-05

All genes were present at a FDR of 0.01 (99% confidence) relative to the vehicle control, DMSO. The list excludes ESTs and other non-annotated sequences.

**Table 2:** Top 40 known genes altered by MS-275 in MC3T3-E1 cells

**Gene**	**Fold Change**	**Welch Test**
**Induced**		
AMP deaminase 3	4.177	1.93E-06
Musculoskeletal, embryonic nuclear protein 1	3.427	1.35E-05
Adenylosuccinate synthetase like 1	3.038	4.20E-05
Amphiregulin	2.710	4.64E-05
Lysyl oxidase	2.697	1.92E-06
Colony stimulating factor 1 (macrophage)	2.694	9.32E-05
Fatty acid binding protein 4, adipocyte	2.518	4.52E-05
Chemokine (C-C motif) ligand 9	2.505	4.00E-05
Solute carrier family 9 isoform 3 regulator 1	2.477	3.63E-06
Sorbitol dehydrogenase 1	2.431	1.89E-07
Troponin C, cardiac/slow skeletal	2.408	6.23E-05
Proline arginine-rich end leucine-rich repeat	2.391	2.05E-05
Glutaminyl-peptide cyclotransferase (glutaminyl cyclase)	2.387	7.24E-05
Inhibitor of DNA binding 1	2.358	1.24E-05
Solute carrier family 40, member 1	2.275	1.04E-04
Glutathione S-transferase, alpha 4	2.268	3.40E-07
Septin 4	2.195	9.94E-05
Voltage-dependent calcium channel gamma subunit-like	2.187	4.78E-05
Immediate early response 3	2.171	4.22E-06
Dimethylarginine dimethylaminohydrolase 1	2.165	1.49E-06
Similar to Normal mucosa of esophagus specific gene 1	2.157	2.91E-06
MAS-related GPR, member F	2.122	1.15E-04
Microtubule-associated protein tau	2.101	9.32E-06
Retinoic acid induced 3	2.095	1.92E-05
High mobility group AT-hook 1	2.088	7.18E-05
A disintegrin-like and metalloprotease with thrombospondin type 1 motif, 5	2.088	7.60E-05
Connective tissue growth factor	2.075	3.62E-05
A kinase (PRKA) anchor protein (gravin) 12	2.036	1.78E-05
Aldehyde dehydrogenase family 3, subfamily A1	2.029	1.25E-04
Galactokinase 2	2.017	2.88E-06
Dystrophia myotonica kinase, B15	2.014	3.81E-05
Thrombospondin 1	2.005	7.11E-05
		
**Suppressed**		
Proline 4-hydroxylase, alpha polypeptide III	-2.006	4.79E-06
Interleukin 1 receptor-like 1	-2.035	4.28E-06
Proteasome (prosome, macropain) subunit, beta type 10	-2.063	2.96E-06
Guanylate nucleotide binding protein 2	-2.284	2.04E-06
Transcription factor AP-2 beta	-2.299	2.27E-05
Sirtuin 1	-2.450	6.21E-05
Adaptor-related protein complex AP-4, sigma 1	-2.484	1.97E-06

All genes were present at a FDR of 0.01 (99% confidence) relative to the vehicle control, DMSO. The list excludes ESTs and other non-annotated sequences.

**Table 3:** Top 10 known genes altered by VPA in MC3T3-E1 cells

**Gene**	**Fold Change**	**Welch Test**
**Induced**		
Glutathione S-transferase, theta 1	2.345	1.73E-06
Glutaminyl-peptide cyclotransferase	2.334	2.36E-05
Sorbitol dehydrogenase 1	2.328	1.91E-06
Procollagen, type XI, alpha 1	2.216	7.84E-06
Solute carrier family 9, isoform 3 regulator 1	2.203	2.33E-06
		
**Suppressed**		
Proteasome subunit, beta type 10	-2.035	3.33E-06
Adaptor-related protein complex AP-4, sigma 1	-2.119	4.39E-07
RIKEN cDNA 2310039E09 gene	-2.759	1.48E-05
Tenascin N	-2.913	8.53E-06
Chemokine (C-X-C motif) receptor 6	-3.728	1.56E-07

All genes were present at a FDR of 0.01 (99% confidence) relative to the vehicle control, DMSO.

**Figure 2:**
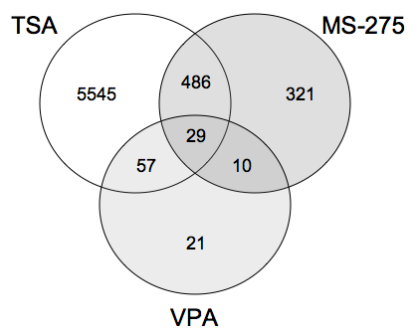
**Venn diagram of differentially expressed genes in TSA, MS-275, or VPA-treated cells**. MC3T3 cells were treated with an HDI or DMSO for 18 hours. Affymetrix GeneChip microarrays and bioinformatics analysis identified genes that were differentially expressed by more than 1.1 fold in HDI-treated cells relative vehicle (DMSO)-treated cells with a FDR of 0.01 (99% confidence).

**Figure 3:**
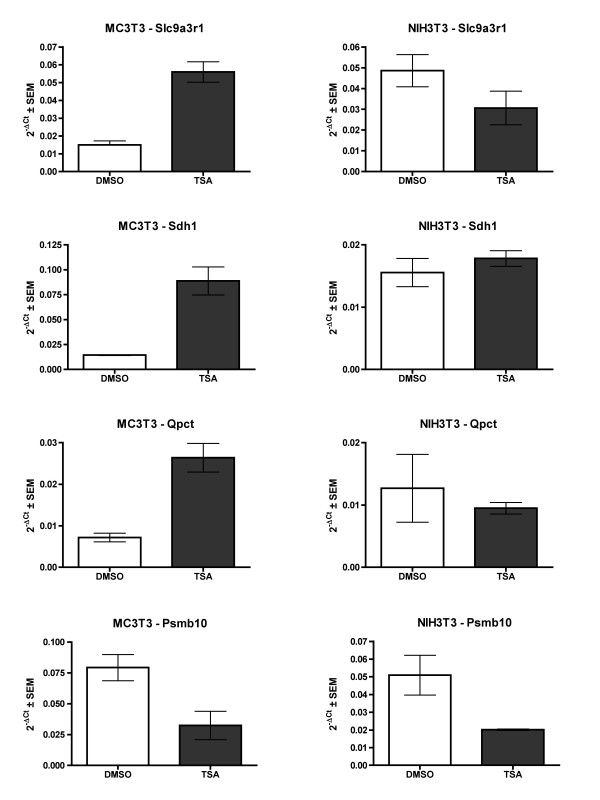
**Specificity of HDI-inducible genes**. MC3T3 and NIH3T3 cells were cultured in osteogenic medium containing TSA or DMSO for 18 hours. mRNAs were isolated, reverse transcribed and amplified using semi-quantitative real-time PCR with primers for Slc9a3r1 (A) or Sdh1 (B), Qpct (C) and Psmb10 (D). Comparative threshold values represent the mean of three samples normalized to actin levels.

To compare the differentially expressed genes in all three HDI treatments relative to DMSO, Venn analysis was performed (Figure 2). Of the 29 genes differentially regulated by all three HDIs, 21 were induced and eight were suppressed (Table 4). Only four of these 29 genes were differentially regulated more than two-fold by each of the three HDIs (Table 5). Three of these four genes were induced; they are solute carrier family 9 isoform 3 regulator 1 (Slc9a3r1), glutaminyl-peptide cyclotransferase (Qpct), and sorbitol dehydrogenase I (Sdh1). These genes are not affected by TSA in NIH3T3 cells, suggesting specificity to osteoblasts (Figure 3). The suppressed gene is proteasome subunit beta type 10 (Psmb10). This gene was also downregulated by TSA in NIH3T3 cells, indicating that it was not specifically targeted in the osteoblasts. When the FDR was changed to 0.05 (95% confidence), four additional genes were altered more than two-fold by each of the three HDIs (Table 5). Three genes (A kinase anchor protein 12 (Akap12), Glutathione S-transferase alpha 4 (Gsta4), and Ral GEF with a PH domain and SH binding motif 2 (Ralgps2)) were induced by the HDIs and one gene, Adaptor-related protein complex AP-4 sigma 1 (Ap4s1), was suppressed.

**Table 4:** Genes differentially regulated by all three HDIs

**Gene Description**	**TSA**	**MS-275**	**VPA**
**Induced**	*Fold Change*		
Thioredoxin interacting protein	6.865	1.530	1.404
Solute carrier family 9, isoform 3 regulator 1	5.565	2.477	2.203
Glutaminyl-peptide cyclotransferase (glutaminyl cyclase)	5.046	2.387	2.334
Sorbitol dehydrogenase 1	4.455	2.431	2.328
Histocompatibility 2, Q region locus 5	4.009	1.634	1.820
Phosphomannomutase 1	3.895	1.902	1.808
RAB3D, member RAS oncogene family	3.717	1.528	1.742
Cbp/p300-interacting transactivator	3.458	1.658	1.321
Protease, serine, 11	3.326	1.836	1.744
Syntaxin 11	2.488	1.942	1.750
Protein phosphatase 1, regulatory (inhibitor) subunit 14c	2.484	1.347	1.830
Lysosomal-associated protein transmembrane 4B	2.366	1.503	1.637
Similar to normal mucosa of esophagus specific gene 1	2.099	2.157	1.930
F-box and leucine-rich repeat protein 16	2.019	1.746	1.561
HIV-1 Rev binding protein	1.953	1.406	1.414
Heat shock 70 kDa protein 5 binding protein 1	1.946	1.358	1.589
Cysteine rich protein 2	1.929	1.847	1.773
Asparagine synthetase	1.597	1.451	1.248
Serum deprivation response	1.485	1.517	1.676
Thymus cell antigen 1, theta	1.419	1.638	1.676
Ribonuclease, RNase A family 4	1.341	1.341	1.529
			
**Suppressed**			
Interleukin 1 receptor-like 1	-1.634	-2.035	-1.831
Low density lipoprotein receptor	-2.487	-1.282	-1.377
Proteolipid protein 2	-2.717	-1.274	-1.233
PHD finger protein 15	-4.100	-1.498	-1.613
Vesicle-associated membrane protein 8	-4.130	-1.417	-1.500
Proteasome (prosome, macropain) subunit, beta type 10	-5.362	-2.063	-2.035
Chromosome condensation 1-like	-7.016	-1.514	-1.721
Glutathione peroxidase 7	-8.216	-1.768	-1.972

All genes were present at a FDR of 0.01 (99% confidence) relative to the vehicle control, DMSO.

**Table 5:** Genes differentially regulated more than two-fold by all three HDIs

**Gene Description**	**TSA**	**MS-275**	**VPA**
**Induced**	*Fold Change*		
A kinase anchor protein 12 (Akap12)	9.5	2.0	2.6
Glutaminyl-peptide cyclotransferase (Qpct)*	5.0	2.4	2.3
Glutathione S-transferase, alpha 4 (Gsta4)	8.5	2.3	3.1
Ral GEF with PH domain and SH3 binding motif 2 (Ralgps2)	2.2	2.0	2.1
Solute carrier family 9, isoform 3 regulator 1 (Slc9a3r1)*	5.6	2.5	2.2
Sorbitol dehydrogenase 1 (Sdh1)*	4.5	2.4	2.3
			
**Suppressed**			
Adaptor-related protein complex AP-4, sigma 1 (Ap4s1)	-4.9	-2.5	-2.1
Proteasome (prosome, macropain) subunit, beta type 10 (Psmb10)*	-5.4	-2.1	-2.0

All genes were present at a FDR of 0.05 (95% confidence) relative to the vehicle control, DMSO. An asterisk (*) indicates genes present at a FDR of 0.01 (99% confidence).

**Figure 4:**
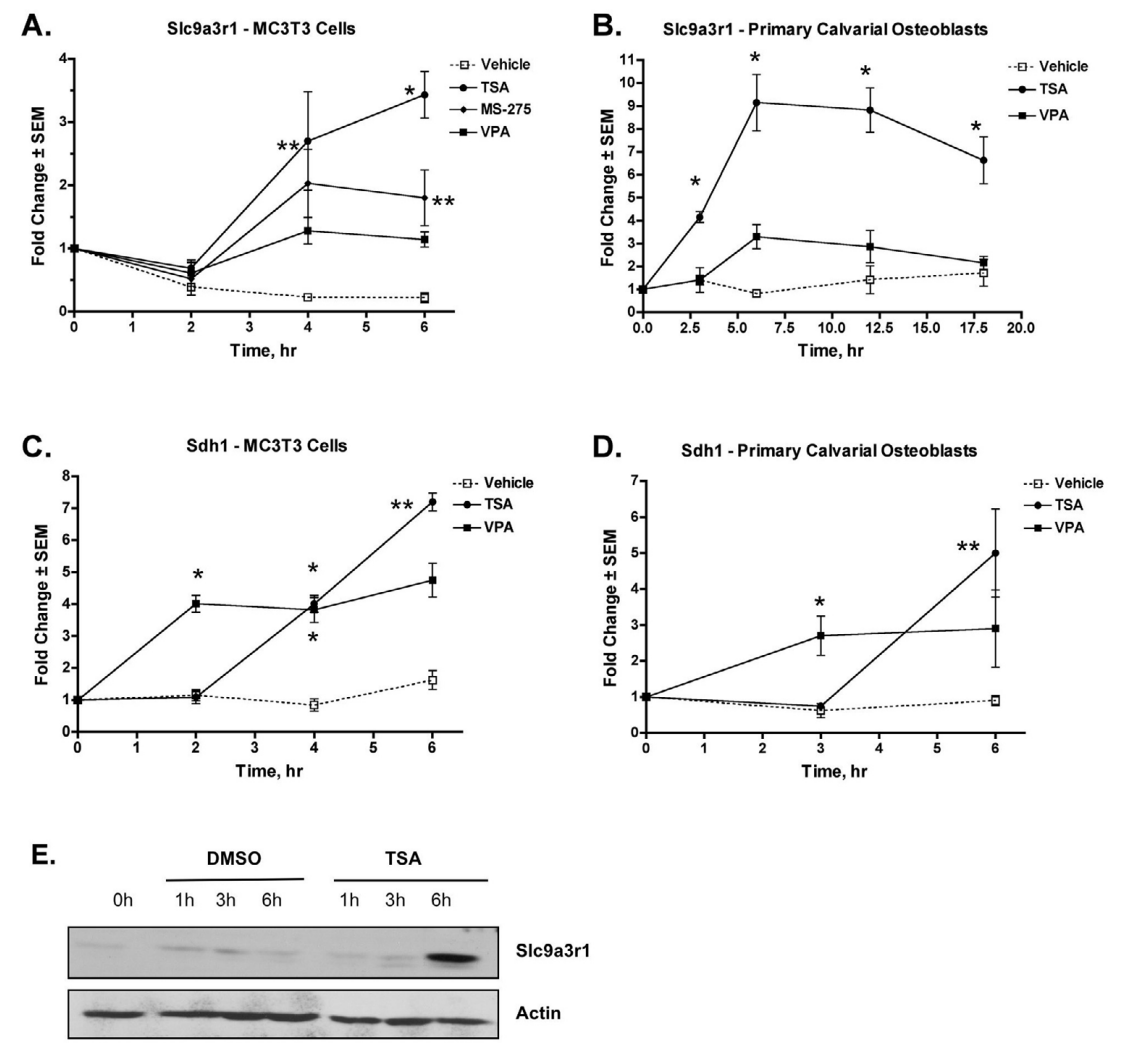
**Verification of Slc9a3r1 and Sdh1 as HDI-inducible genes**. MC3T3 (A and C) or primary calvarial osteoblasts (B and D) were cultured in osteogenic medium containing the indicated HDI or DMSO. mRNAs were isolated at various times over a 6 or 18 hour period and subjected to quantitative real-time PCR with primers for Slc9a3r1 (A and B) or Sdh1 (C and D). Values are relative to those obtained from DMSO-treated samples at each time point and represent the mean of three samples. For (A-D), * denotes a statistically significant change of p < 0.01 and ** denotes p < 0.05 by one-way ANOVA of the HDI-treated sample versus the DMSO-treated sample at that time point. (E) C2C12 cells were cultured in osteogenic medium and 20 nM TSA for the indicated times. Slc9a3r1 protein levels were determined by immunoblotting.

**Figure 5:**
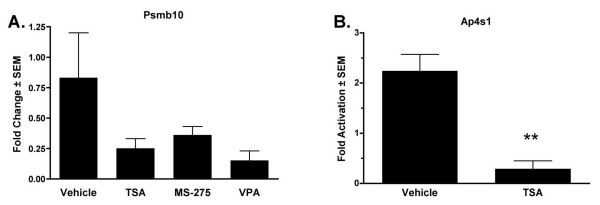
**Verification of Psmb10 and Ap4s1 as HDI-suppressed genes**. Primary calvarial osteoblasts were cultured in osteogenic medium containing the indicated HDI or DMSO. mRNAs were isolated after 18 hours and subjected to quantitative real-time PCR with primers for Psmb10 (A) or Ap4s1 (B). Values are relative to those obtained from DMSO-treated samples at each time point and represent the mean of three samples. ** denotes p < 0.05 by one-way ANOVA of the HDI-treated sample versus the DMSO-treated sample at that time point.

The temporal regulation of several identified genes was determined by quantitative real-time PCRs using mRNAs isolated from independent cultures of MC3T3 cells or primary murine calvarial osteoblasts. Consistent with the GeneChip analysis, TSA was a more potent inducer of Slc9a3r1 than MS-275 or VPA in both MC3T3 cells (Figure 4A) and primary osteoblasts (Figure 4B). Slc9a3r1 induction was detectable at both the RNA and protein levels as early as 4 to 6 hours after HDI exposure (Figure 4B and 4E). Similar effects were observed with Sdh1 mRNA in both MC3T3 (Figure 4C) and primary osteoblasts (Figure 4D) except VPA induced a more rapid increase in Sdh1 expression than TSA. The induction of Akap12 was also confirmed in primary osteoblasts (data not shown). The suppression of Psmb10 and Ap4s1 was confirmed in primary osteoblasts (Figure 5).

### Regulation of growth factor and growth factor receptor genes by HDIs

We previously demonstrated that HDIs induced the differentiation rate of MC3T3 cells and primary calvarial osteoblasts [18]. Therefore we analyzed the microarray data sets for growth factor and growth factor receptor genes that were differentially regulated by MS-275, VPA and TSA with greater than 95% confidence. Of the eight genes identified, only three were induced by all of the HDIs (Table 6). Amphiregulin, a preostoblast growth factor [28], was most consistently and highly increased. Brain-derived neurotrophic factor and the Wnt/Norrin receptor, Frizzled 4 (Fz4), were also stimulated by HDIs. In contrast, Fz1, an antagonist of osteoblast growth [29], was suppressed by HDIs. Quantitative real-time PCR analyses verified the differential regulation of several of these genes in primary osteoblasts (Table 6) and MC3T3 cells (data not shown).

**Table 6:** Growth factor and growth factor receptor genes regulated by HDIs

	**MC3T3 Cells Microarray**			**Primary Osteoblasts qRT-PCR**	
	
**Growth factor/receptor**	**TSA**	**MS-275**	**VPA**	**TSA**	**VPA**
	*Fold Change*				
Amphiregulin	7.10	2.74	1.82	24.8	2.56
Brain-derived neurotrophic factor	2.51	1.66	1.77	ND	ND
Fibroblast growth factor 7	-5.80	-1.10	-1.17	-29.9	1.05
Frizzled 1	-2.17	-1.25	-1.24	-2.53	-1.44
Frizzled 4	2.23	1.37	1.53	1.79	2.57
Interleukin 1 receptor-like 1	-1.63	-2.04	-1.83	-52.1	1.04
Interleukin 17 receptor c	-2.62	-1.50	-1.43	ND	ND
Smoothened	-2.60	-1.53	-1.17	ND	ND

Cells were incubated with HDIs for 18 hr. All genes were present at a FDR of 0.05 (95% confidence) relative to the vehicle control, DMSO. ND, not determined.

## Discussion

HDIs are potential bone anabolic agents due to their ability to promote osteoblast maturation and osteo-progenitor expansion [18,21,22,24,25]. We previously showed that the addition of HDIs to MC3T3 cell cultures for three or six days accelerated the expression of known early and late osteoblast differentiation genes [18]. To identify genes affected early by the HDIs, we differentiated and treated MC3T3-E1 cells with three HDIs or vehicle for 18 hours and analyzed gene expression profiles with Affymetrix microarray gene chips. Relative to vehicle-treated cells, TSA altered the expression of 6117 genes; MS-275 altered 846 genes; and VPA changed 117 genes. The large differences between the numbers of genes affected by the HDIs may be due to differences in their potencies, half-lives and/or specificities. Nanomolar concentrations of TSA inhibit the activities of all HDACs [7]. Likewise, MS-275 is effective at nanomolar concentrations; however, it does not inhibit HDAC8 [9]. In contrast, VPA is effective at millimolar concentrations and specifically inhibits class I and II HDACs with the exceptions of HDAC6 and HDAC10 [8]. Tables 2, 3, 4 list the genes most differentially regulated by each HDI. As indicated in the Venn diagram (Figure 2), some genes on these lists are affected by just one HDI. These genes might appear just in one group because we only assayed one time point; however, it is possible that each HDI affects a distinct subset of genes, some of which might not affect osteoblast differentiation. These differences could affect the suitability of an HDI for a specific application.

All three HDIs (TSA, MS-275 and VPA) used in this study accelerate MC3T3 terminal differentiation and induce alkaline phosphatase activity in calvarial organ cultures [18]. Genes significantly altered by all three HDIs may represent a core group of genes that are responsible for initiating the acceleration of osteoblast differentiation. Of note, well-known osteoblast genes, such as osteocalcin, did not appear in our results. It is likely that the time of analysis (18 hours) was too early to see differential effects on these genes. Of the most differentially regulated genes (Table 5), Slc9a3r1 is the most likely to have roles in osteoblast proliferation and/or differentiation.

Slc9a3r1, also known as Na+/H+ exchanger regulatory factor (NHERF) or ezrin binding protein 50, is an apical membrane phosphoprotein that links membrane proteins with cytoplasmic proteins to regulate actin cytoskeletal reorganization [30]. Slc9a3r1 interacts with numerous signaling proteins [31], including the G-protein coupled receptor for parathyroid hormone [32], and the canonical Wnt signal transducer, β-catenin [33]; thereby implicating its potentially important role in osteoblast maturation. *Slc9a3r1 (NHERF-1) *deficient mice develop renal phosphate wasting, but the majority of female mice also had a 25–30% reduction in bone mineral density and a 40% decrease in bone mineral content with multiple fractures [34]. We found that overexpression of Slc9a3r1 in osteoblasts by adenoviral transduction was not sufficient to drive osteoblast differentiation (data not shown). These data indicate that NHERF-1 contributes to bone homeostasis but is not sufficient to promote osteoblast maturation.

Another differentially regulated gene is proteasome subunit beta type 10 (Psmb10). Also known as Lmp10 and MECL1, Psmb10 was suppressed by HDIs in both MC3T3 osteoblasts and NIH3T3 cells. Psmb10 is one of ten proteolytically-active beta subunits of the 20S core complex within the 26S proteosome [35]. Ubiquitin-mediated proteasomal degradation has an established role in osteoblasts [36,37]. Compounds that bind to the 20S proteasome beta subunits, inhibit the ubiquitin-proteasome pathway and stimulate bone formation in vitro and in vivo [36]. Therefore, the decreased expression of Psmb10 upon HDI treatment is consistent with the observed acceleration of osteoblast differentiation. The specific role HDAC activity plays in the regulation of Psmb10 expression is unclear. The non-specific decreased expression of this gene upon HDI treatment suggests that the Psmb10 promoter is probably not directly regulated by HDACs but rather through a repressor whose expression is sensitive to HDAC activity.

We previously reported that HDIs induce the proliferation and/or survival of MC3T3 cells and primary calvarial osteoblasts but do not affect cell cycle progression [18]. Examination of the microarray gene lists for growth factor and growth factor receptor genes affected by HDIs identified eight genes that could influence osteoblast growth, but only three were increased. Amphiregulin was highly induced by HDIs (Table 6). Amphiregulin binds EGF receptors and is a potent growth factor for preosteoblasts [28]. Brain derived neurotrophic factor mRNA was also increased by HDIs, but its effects on osteoblast growth have not been determined. Two Frizzled genes were also regulated by HDIs. Frizzled-4 was increased by HDIs, while Frizzled-1 levels were suppressed. Frizzled-4 is a receptor for Norrin [38] and presumably Wnts, which promote osteoblast progenitor proliferation and survival [39]. In contrast, murine Frizzled-1 is a negative regulator of bone formation [29]; thus its suppression is consistent with differentiation promotion by HDIs. No other components of the Wnt signaling pathway were significantly regulated by all HDIs at the 18-hour time point.

## Conclusion

We identified many osteoblast genes whose expression levels are altered by HDIs. All genes we have tested to date are similarly differentially regulated in both MC3T3-E1 cells and primary murine calvarial osteoblasts. These data improve our understanding of how HDIs promote osteoblast differentiation by identifying genes that are altered within the first 18 hours of HDAC inhibition.

## Methods

### Cell culture

MC3T3-E1 preosteoblasts were plated at 2 × 10^5 ^cells per 6-cm plate and 4 × 10^4 ^cells per well of a 12-well plate and differentiated in Minimal Essential Medium (Invitrogen, Carlsbad, CA) containing 10% FBS (Invitrogen and Cambrex Bioscience/Lonza, Basel. Switzerland), 100 U/ml penicillin, 100 μg/ml streptomycin, 50 μg/ml ascorbic acid, 10 mM β-glycerol phosphate and one of the following compounds: 20 nM TSA (Sigma, St. Louis, MO), 500 nM MS-275 (Calbiochem, San Diego, CA), 500 mM VPA (Sigma), or DMSO (vehicle). During the differentiation assay, the osteogenic medium was replaced every three days with the HDIs or vehicle added only at day 0. C2C12 cells and NIH3T3 fibroblasts were maintained Dulbecco's modified Eagle's medium containing 10% FBS, 100 U/ml penicillin and 100 μg/ml streptomycin. Primary calvarial osteoblasts were isolated as previously described [18]. Briefly, calvaria from newborn CD1 mice were collected and sequentially rinsed in Hank's Balanced Salt Solution (Invitrogen) and serum-free Minimal Essential Medium (Invitrogen). Calvaria were digested into a single cell suspension in serum-free alpha- Minimal Essential Medium containing 2 mg/ml collagenase and 0.25% trypsin. Cells were washed, plated at 2 × 10^5 ^cells/10 cm plate and incubated with HDI as described above.

### RNA, cDNA, and biotin-labeled cRNA preparation

To ensure statistical significance of microarray analyses, quadruplicate cultures of MC3T3-E1 cells were incubated in osteogenic medium containing DMSO or HDIs. Total RNA was isolated from each MC3T3-E1 cell culture and primary calvarial osteoblasts with Trizol reagent (Invitrogen). For preparation of GeneChip samples, biotin-labeled cRNA was prepared from each of the quadruplicate cultures according to the Affymetrix protocol. Briefly, RNA was denatured at 70°C with T7-oligo (dT) primer and reverse transcribed using Superscript II at 42°C for 1 hour. Second strand cDNA synthesis was performed by incubating first strand cDNA with *Escherichia coli *DNA polymerase I, *E. coli*. DNA ligase, RNase H, and dNTPs for 2 hours at 16°C. Biotin-labeled cRNA was prepared from double-stranded cDNA using the Affymetrix GeneChip IVT Labeling kit (Affymetrix, Santa Clara, CA), then purified and fragmented using the Affymetrix Sample Cleanup Module.

### Affymetrix genechip

Hybridization of the biotinylated-cRNA to the Affymetrix GeneChip Mouse Genome 430 2.0 Array was performed by the BioMedical Genomics Center's microarray facility at the University of Minnesota using Affymetrix Genechip^® ^Hybridization Oven 640 and Fluidics Station 450. For each condition, four chips were hybridized. Scanning of the chips to detect and quantify hybridization signals was done with the Affymetrix Genechip^® ^Scanner 3000. The gene expression data were preprocessed and analyzed by the University of Minnesota Cancer Center Informatics Shared Resource. Raw data measurements were imported into Genedata Expressionist^® ^Refiner (EPro1.0.32) for overall chip hybridization quality assessment, correction and condensation of probe sets intensity values. Robust Multichip Average (RMA) method was applied for global background subtraction and cross array normalization [40]. Processed expression data were imported into Genedata Expressionist Analyst. General assessment of the data distribution was performed with Principle Component Analysis, boxplot and log-log data plots. Following LOESS normalization, differential expression measures were calculated by Welch test comparisons of control DMSO (vehicle) versus each HDAC inhibitor. Significant genes from each comparison were selected using the Benjamini-Hochberg method to control for a maximum false discovery rate (FDR) of 95% or 99% as indicated. Values for fold change were calculated and genes demonstrating a net change in gene expression of two fold or greater were selected for further analysis.

### Quantitative real-time PCR

Total RNA was isolated from MC3T3-E1, NIH3T3, and primary murine calvarial cells with Trizol reagent (Invitrogen). RNA (1 μg) was reverse transcribed to cDNA with the Invitrogen Superscript Kit. cDNA was amplified with the Qiagen Quantitect SYBR Green RT-PCR kit using gene specific primers (Table 7) in a MyIQ Single Color Real-Time PCR Detection System (BioRad). Quantification and normalization to actin amplicons were performed as previously described [26]. Statistical analyses were performed with Prism 4.0 (GraphPad Software, San Diego, CA).

**Table 7:** Primer sequences used for PCRs

Gene	Strand	Primer Sequences	Annealing Temp (°C)	Product Length (bp)
Akap12	F	5' GCCAGTGAAGAACATGAGCAG	56	160
	R	5' CTATGCAATCTGCTTTGTCTTGG		
Ap4s1	F	5' GTACTTCAGCCGAGTGAGTG	56	202
	R	5' CTCTTCCTTGGCCTTCACAG		
Amphiregulin	F	5' GACTCACAGCGAGGATGACAAG	56	249
	R	5' GCTTGGCAATGATTCAAC		
Fibroblast growth factor 7	F	5' CAGTTTGGAAAGAGCGACGAC	56	170
	R	5' GGCAGGATCCGTGTCAGTATC		
Frizzled-1	F	5' CAAGGTTTACGGGCTCATGTAC	56	180
	R	5' GTAACAGCCGGACAGGAAAATG		
Frizzled-4	F	5' CTGCAGCATGCCTAATGAGAG	56	185
	R	5' CGTCTGCCTAGATGCAATCA		
Interleukin 1 receptor-like 1	F	5' CCAGCCAGAGTGGAAGACTC	56.9	189
	R	5' CAGGTCAATTGTTGGACACG		
Psmb10	F	5' CTTTACTGCCCTTGGCTCTG	56.9	168
	R	5' GTGATCACACAGGCATCCAC		
Qpct	F	5' GAGGAGGCACTGAAGGAGTG	53	162
	R	5' GAAGATCGGACTGGATGCTC		
Sdh1	F	5' CTGCCGATTCTACAAGCACA	56.9	183
	R	5' AGCAAAGTGACCATCCCAAC		
Slc9a3r1	F	5' CAAAGTGTCCCCTCACCAGT	56.9	209
	R	5' AATGAACCCAAGATGCCAAG		

### Alkaline phosphatase activity assay

Alkaline phosphatase activity was measured on the indicated days as previously described [26]. Briefly, MC3T3-E1 cells were washed in PBS, lysed in 0.2% NP-40 and 1 mM MgCl_2_, sonicated, and spun at 3000 rpm for 15 minutes at 4°C. The supernatants were added to a reaction solution containing 0.6 M 2-amino-2-methyl-1-propanol, 2.4 mM MgCl_2_, and 9.6 mM p-nitrophenyl phosphate and incubated at 37°C for 30 minutes, at which time the reactions were stopped with 2N NaOH and the absorbance read at 410 nm. Alkaline phosphatase activity was normalized to protein content. Protein content was determined using the DC Protein Assay system (BioRad).

### Immunoblotting

Cell lysates were prepared by rinsing cultures with PBS prior to lysing the cells on ice for 5 minutes in modified RIPA buffer (50 mM Tris-HCl, pH 7.4, 150 mM sodium chloride, 1% NP-40, 0.25% sodium deoxycholate, 1 mM EDTA) supplemented with complete protease inhibitor tablets (Roche, Basel, CH). Crude lysates were sonicated and cleared by centrifugation at 10,000 rpm at 4°C. Total protein was quantified using the detergent-compatible protein assay (BioRad, Hercules, CA). Protein (50 μg) was resolved by SDS-PAGE on an 8% gel and transferred to Immobilon-P membranes (Millipore, Billerica, MA). Membranes were sequentially blotted with antibodies against NHERF1/EBP50 (ab3452; Abcam, Cambridge, MA) and HRP-conjugated secondary antibodies (Sigma). Proteins were visualized using ECL-Plus chemiluminescent substrate (G.E. Health Systems).

## Competing interests

The author(s) declares that there are no competing interests.

## Authors' contributions

TMS generated the microarray data and drafted the manuscript. AKN verified differentially expressed genes. RS and AFL performed bioinformatics analysis of the microarray data. JJW provided support, direction, and oversight of the experiments and revised the final manuscript. All authors have read and approved the final manuscript.
